# Previous High-Intensity Breastfeeding Lowers the Risk of an Abnormal Fasting Glucose in a Subsequent Pregnancy Oral Glucose Tolerance Test

**DOI:** 10.3390/nu16010028

**Published:** 2023-12-21

**Authors:** Sarah J. Melov, James Elhindi, Lisa White, Justin McNab, Vincent W. Lee, Kelly Donnolley, Thushari I. Alahakoon, Suja Padmanabhan, N. Wah Cheung, Dharmintra Pasupathy

**Affiliations:** 1 Reproduction and Perinatal Centre, Faculty of Medicine and Health, The University of Sydney, Sydney, NSW 2006, Australia; james.elhindi@sydney.edu.au (J.E.); justin.mcnab@sydney.edu.au (J.M.); dharmintra.pasupathy@sydney.edu.au (D.P.); 2 Westmead Institute for Maternal and Fetal Medicine, Women’s and Newborn Health, Westmead Hospital, Westmead, Sydney, NSW 2145, Australia; indika.alahakoon@health.nsw.gov.au; 3 Women’s Health Maternity, Blacktown and Mt Druitt Hospitals, Blacktown, NSW 2148, Australia; lisa.white@health.nsw.gov.au; 4 Faculty of Medicine and Health, The University of Sydney, Sydney, NSW 2006, Australia; vincent_lee@wmi.usyd.edu.au (V.W.L.); wah.cheung@sydney.edu.au (N.W.C.); 5 Department of Renal Medicine, Westmead Hospital, Westmead, Sydney, NSW 2145, Australia; 6 Consumer Representative, Western Sydney Local Health District, Sydney, NSW 2151, Australia; 7 Department of Diabetes and Endocrinology, Westmead Hospital, Westmead, Sydney, NSW 2145, Australia; suja.padmanabhan@sydney.edu.au

**Keywords:** breastfeeding, cardiovascular disease, diabetes, gestational diabetes mellitus, lactation, pregnancy, type 2 diabetes mellitus

## Abstract

Breastfeeding is associated with reduced lifetime cardiometabolic risk, but little is known regarding the metabolic benefit in a subsequent pregnancy. The primary aim of this study was to investigate the association between breastfeeding duration and intensity and next pregnancy oral glucose tolerance test (OGTT) results. A retrospective cohort study was conducted from March 2020 to October 2022. All multiparous women who met inclusion criteria and gave birth during the study period were eligible for inclusion. Analysis was stratified by risk for gestational diabetes (GDM). High GDM risk criteria included previous GDM and BMI > 35 kg/m^2^. The association between breastfeeding duration and high-intensity breastfeeding (HIBF) and subsequent pregnancy OGTT were assessed with multivariate logistic models adjusted for statistically and clinically relevant covariables. There were 5374 multiparous participants who met the inclusion criteria for analysis. Of these, 61.7% had previously breastfed for >6 months, and 43.4% were at high risk for GDM. HIBF was associated with 47% reduced odds of an abnormal fasting glucose in a subsequent pregnancy OGTT (aOR 0.53; 95%CI 0.38–0.75; *p* < 0.01). There was no association between HIBF and other glucose results on the OGTT. Women who smoked were least likely to breastfeed at high intensity (aOR 0.31; 95%CI 0.21–0.47; *p* < 0.01). South Asian women had 65% higher odds of HIBF than women who identified as White/European (aOR 1.65; 1.36–2.00; *p* < 0.01). This study highlights the importance of exclusive breastfeeding to potentially reduce the prevalence of GDM and may also translate into long-term reduction of cardiometabolic risk.

## 1. Introduction

Women who breastfeed for a greater duration and more exclusively have a reduced lifetime risk of type 2 diabetes as well as an improved cardiometabolic profile [1,2,3]. Breastfeeding is supported by groups such as the World Health Organization (WHO), who recommends exclusive breastfeeding as critical for infant health for the first six months of life and continued breastfeeding to age two and beyond [4]. Further to the well- established infant health advantages of breastfeeding, there are globally recognized economic savings linked to the reduction in maternal and infant mortality and morbidity as well as environmental benefits associated with breastfeeding [5,6]. However, exclusive breastfeeding rates remain obstinately low, with little chance that the WHO global target of 70% exclusive breastfeeding during the first six months will be met by the target year of 2030 [7].

Gestational diabetes (GDM) rates vary depending on diagnostic criteria and population; estimates are between 4% and 28%, with a documented rising prevalence [8,9]. Women with GDM have a lifetime twofold increased risk of cardiovascular disease (CVD) and an estimated six- to tenfold maternal future risk of type 2 diabetes [10,11]. The rise in global mortality from non-communicable diseases (NCDs) is a global health crisis, recognized by the United Nations’ Sustainable Development Goals to reduce NCD-preventable mortality by one-third by 2030 [12]. To help meet these targets, urgent preventive measures are required to reduce the incidence of GDM and thus maternal and infant type 2 diabetes risk. Various strategies have been adopted, aimed at lowering the prevalence of GDM and type 2 diabetes, focusing principally on lifestyle intervention, with inconsistent findings [13,14]. However, breastfeeding as a type 2 diabetes prevention measure has been inadequately supported despite evidence of reducing the relative risk of type 2 diabetes risk by 50% [15]. The impact of previous breastfeeding on GDM risk in the next pregnancy is largely unknown. Breastfeeding studies investigating diabetes risk have concentrated on changes in postpartum cardiometabolic markers, such as lipids or OGTT results during the early postpartum period and ongoing type 2 diabetes incidence [16,17]. We are not aware of any studies that have investigated breastfeeding and next pregnancy glycaemic metabolism except our pilot study [18]. The pilot study was undertaken in a selected high-risk population (previous GDM) and found that both duration and exclusivity of breastfeeding were associated with improved glucose levels on a subsequent pregnancy OGTT [18].

For multiparous women, it is important to understand previous breastfeeding history to assess cardiometabolic risk and provide an opportunity for lactation support interventions. In recognition of the importance of breastfeeding for both infant and maternal health, the Sydney BLISS check was introduced to improve antenatal breastfeeding support in our health district. The BLISS tool was developed as part of our pilot study [18] and is now used in routine antenatal clinical care to assess breastfeeding during the first 12 weeks postpartum (‘fourth trimester’) after a woman’s previous pregnancy [19].

In this study, we aimed to build on the findings of our pilot study to investigate the association between previous pregnancy breastfeeding intensity and duration and OGTT results in a subsequent pregnancy for an unselected population. The secondary aim was to understand breastfeeding patterns to identify specific groups of women at risk for suboptimal breastfeeding, who may then be at increased risk for cardiometabolic disease.

## 2. Materials and Methods

We conducted a retrospective cohort study of women booked to give birth in the Western Sydney Local Health District (WSLHD), Sydney, Australia between March 2020 and October 2022. The study period was determined from the implementation into routine clinical care of the antenatal breastfeeding history and the triaging assessment tool, the Sydney BLISS check, which was introduced to improve breastfeeding support.

**Figure 1:**
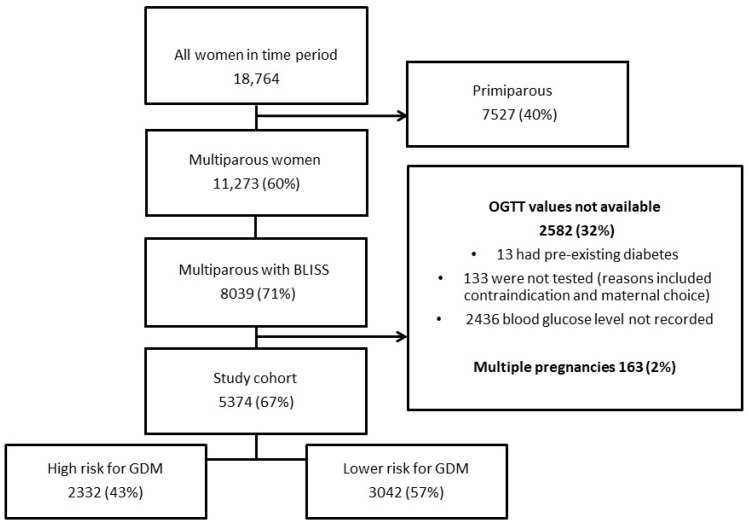
Flow Chart.

The population is culturally and linguistically diverse, with approximately 58% of women who give birth in the district born in a non-English speaking country [20]. The WSLHD has three maternity care hospitals, with approximately 10,000 births per year [20]. Hospitals in the district were included in the study cohort after >50% of multiparous women at the hospital received midwifery breastfeeding assessment by the BLISS check at booking. The study period for the health district hospitals were Hospital 1: 1 March 2020–31 October 2022; Hospital 2 and 3: 1 January 2022–31 October 2022 (see Supplementary File S1: Figure S1). All women with a singleton pregnancy ≥ 20 weeks’ gestation and who had had a previous live birth were included in the study. Exclusion criteria included multiple pregnancy, no BLISS check or OGTT result available, or previous diagnosis of type 1 or type 2 diabetes (Figure 1). Incomplete OGTT results were reviewed; reasons included inadequate documentation, patient unable to tolerate glucose drink and patient declined testing (Figure 1).

### 2.1. Measures and Data Source

The data source was the electronic maternity database eMaternity, providing routinely collected information during pregnancy, including medical and obstetric history. eMaternity in WSLHD includes Sydney BLISS check data.

### 2.2. Breastfeeding Measures

The Sydney BLISS tool was embedded into eMaternity WSLHD on 1 March 2020. COVID-19 interrupted the implementation of the assessment tool at the two smaller of the three district hospitals until 2022. The BLISS check was designed by a panel of lactation experts and assesses breastfeeding intensity (exclusivity) in the first three months after a woman’s last pregnancy [18]. Intensity is the ratio of breastfeeding to infant formula feeding, with high-intensity breastfeeding (HIBF) being mostly or exclusively breastfeeding. The BLISS assessment aims to identify a history of breastfeeding issues via a standardized scoring system to triage women for antenatal lactation support. The score is auto-generated as part of routine booking electronic data collection and care. The BLISS check also collects information on total duration of any breastfeeding and the reasons for stopping breastfeeding. Women at booking are routinely offered a telehealth antenatal lactation clinic referral for a low BLISS score or if other breastfeeding issues are identified. HIBF is determined as a BLISS score of ≥19 as optimal maternal breastfeeding intensity and equates to approximately >70% breastfeeding to formula in the first three months postpartum [18]. Lower intensity breastfeeding (LIBF) reflects less optimal breastfeeding and a BLISS score of <19 [18]. The Sydney BLISS assessment tool is administered by the booking midwife (see Supplementary File S2: Sydney BLISS check). We dichotomized breastfeeding analysis to ≤6 months and >6, as this is the recommended time to exclusively breastfeed prior to introduction of family foods [4]. Six months is also a recognized population benchmark time period for assessing any breastfeeding [21].

### 2.3. Gestational Diabetes Measures

At the study hospitals all women are routinely screened for GDM via a one-step OGTT at 24–28 weeks’ gestation, or earlier when clinically indicated. The 24–28 weeks’ gestation results in this study were used for analysis if an early test was administered and repeated at 24–28 weeks’ gestation. The early gestation results were used if no other OGTT was administered. Pathology results are routinely reviewed by the treating clinician and results entered in electronic records. Diagnosis of GDM in this study used the International Association of Diabetes and Pregnancy Study Group (IADPSG) criteria [22]. IADPSG thresholds were as follows: one or more values ≥ thresholds of fasting plasma glucose of 5.1 mmol/L, 1 h 10.0 mmol/L and/or a 2 h plasma glucose level of 8.5 mmol/L following a 75 g OGTT. High risk for GDM for the study cohort was defined as per the Australian Diabetes in Pregnancy Society v2 2014 criteria: previous GDM, maternal age ≥ 40 years, family history of diabetes, BMI >35 kg/m^2^, previous baby with birth weight >4500 g or >90th centile, polycystic ovarian syndrome and current use of corticosteroids or antipsychotics [23].

### 2.4. Body Mass Index Measure (BMI)

The BMI variable is a perinatal data collection (PDC) data point collected for all women who birth in the state of New South Wales to assess pregnancy BMI trends [20]. In our health district, it is collected in the eMaternity database. The height is collected by the midwife at first pregnancy hospital booking visit, and the weight is a pre-pregnancy weight provided by the patient. This weight is ‘sense-checked’ by a current weight taken by the midwife at booking visit. Fidelity of data collection is ensured by frequent review by the data custodian.

### 2.5. Demographic Measures

Socioeconomic status (SES) is defined by place (suburb) of residence estimate and derived from information provided during the Australian census (2016), which informs the Index of Relative Socioeconomic Disadvantage (IRSD) [24]. Age was calculated at the time of booking for the current pregnancy. Ethnicity was self-assigned by women at booking and does not equate to migrant status. We acknowledge that ethnicity may be also described as race; however, women are asked to self-identify their ethnicity at hospital admission, therefore we are reporting this variable as ethnicity. Women who are migrants provide details of years lived in Australia and are of varied ethnicities.

### 2.6. Statistical Analysis

Statistical analyses were completed in Stata SE Version 14.2 and R Studio Version 4. Hypotheses were conducted at a significance level of 0.05 with a two-sided alternative.

Breastfeeding intensity and breastfeeding duration were considered co-primary exposures of interest. Breastfeeding duration was considered a linear continuous variable. Our models were adjusted for baseline variables measured at pregnancy booking; maternal age, ethnicity, migrancy, SES, BMI, parity, history of mental illness, history of hypertension, history of GDM and smoking during pregnancy. GDM diagnosis was assessed with a logistic regression model. Abnormal glucose tolerance via routine OGTT is a set of up to three binary outcomes (fasting, one-hour and two-hour) from repeated measurements from the same woman. Therefore, a generalized estimating equation (GEE) model, equipped with a logistic link function and first-order autoregressive correlation structure for within woman covariance, was implemented. Odds ratios, 95% confidence intervals and *p* values were reported.

To address the secondary aim of our study, maternal characteristics associated with breastfeeding intensity and duration were assessed. In each case, a logistic regression model was implemented. As an outcome, breastfeeding duration was dichotomized to breastfeeding >6 months versus ≤6 months. A priori variables were decided on as possible confounders for each exposure of interest and reported individually (see Supplementary File S3) [25]. Fasting blood glucose results were further investigated, grouped by Hyperglycaemia and Adverse Pregnancy Outcomes (HAPO) study blood glucose septiles and divided into HIBF or lower intensity breastfeeding plots with separation of curves analysed for significance [22]. Odds ratios, 95% confidence intervals and *p* values were reported.

No imputation was made for missing data, as missingness of the primary independent variable was plausibly missing not at random. We comment on the extent of characteristic differences that reflect hypothesized associations with mechanisms of missingness in Supplementary File S4.

## 3. Results

### 3.1. Cohort Characteristics and Exposure

During the study period, there were 11,273 multiparous women booked at the study hospitals. There were 5374 (67%) participants who met the inclusion criteria (Figure 1). The mean age was 32.4 (SD 6.8) years, and there were 981 patients (18.3%) diagnosed with GDM. Of all participants, 4074 (75.8%) were documented to be HIBF, 3558 (66.2%) had exclusively breastfed at 3 months, and 3222 (61.7%) of the total cohort breastfed for >6 months. The median duration of breastfeeding was 9 months (IQR 4–16).

Women classified in the HIBF group via the Sydney BLISS check breastfed for a median duration of 12 months (IQR 6–18, *n* = 4074, 75.8%) compared to those in the lower intensity group, who had a median duration of 3 months (IQR 1–7 months, *n* = 1300, 24.2%; *p* <0.01) (see Supplementary file S5: Figure S2). There were 2332 (43%) of the total cohort who were at high risk for GDM (Figure 1). There was no difference in the breastfeeding duration or intensity for women in either the high- or low-risk groupings for GDM (Table 1).

There was a graduated decline in the median breastfeeding duration associated with lower age groupings (Table 1). Women <25 years old had the lowest median duration of breastfeeding (6 months: IQR 2–13) compared to the other age groups; women aged >39 years had the longest median duration (12 months; IQR 6–18). This graduated decline with younger age groups was also evident for HIBF (Table 1).

Australian-born women had a lower median duration of breastfeeding (7 months; IQR 3–14) compared to all migrants. Participants who had lived in Australia >10 years breastfed for a shorter duration than newer migrants (9 versus 12 months) (Table 1). Two factors were associated with the lowest median duration of breastfeeding: current smoking status (4 months; IQR 2–12) and women who identified as Aboriginal or Torres Strait Islander (4 months; IQR 2–9) (Table 1). For previous pregnancy complications, preterm birth was the only factor associated with shorter duration of breastfeeding (7 versus 10 months; *p* = 0.003), and this also was a factor for less intensity (67.9% versus 76.4%; *p* < 0.001) (Table 1). Other factors associated with duration and HIBF are detailed in Table 1.

### 3.2. Primary Aim: Association between Breastfeeding and OGTT Results in a Subsequent Pregnancy

Compared to women who breastfed at a lower intensity, women who had breastfed at high intensity in the first three months after their previous birth had a 47% reduced odds of an abnormal fasting blood glucose on the OGTT in their subsequent pregnancy (aOR 0.53; 95%CI 0.38–0.75; *p* < 0.01). The association between HIBF and improved fasting glucose was greater in women at a lower risk of GDM, with a 52% reduced odds of abnormal fasting blood glucose (aOR 0.48; 95%CI 0.27–0.86, *p* = 0.01) and 45% reduced odds for the women in the high-risk group (aOR 0.55; CI 0.36–0.83, *p* = 0.01) (Table 2). There were no associations between the OGTT results at one and two hours with HIBF or duration of breastfeeding (Table 2).

HIBF was not associated with diagnosis of GDM in all women (aOR 0.91; 95%CI 0.75–1.10, *p* = 0.34); women at reduced risk of GDM diagnosis (aOR 1.02; 95%CI 0.75–1.37, *p* = 0.92) or at high risk of GDM (aOR 0.84; 95%CI 0.65–1.08, *p* = 0.18) (Table 2). Breastfeeding duration was not associated with risk for GDM diagnosis overall (aOR 1.01; 95%CI 0.99–1.02, *p* = 0.06) or in the different risk categories (Table 2).

When fasting blood glucose results were viewed as a continuous graph for the two groups of HIBF and lower intensity, separation of groups occurred at the AIDPSG blood glucose cut-off 5.1 mmol/L, displaying HIBF participants with lower fasting blood glucose (*p* = 0.01) (see Supplementary File S6: Figure S3).

### 3.3. Secondary Aim: Factors Associated with Both Reduced High-Intensity Breastfeeding Postpartum and Breastfeeding >6 Months

There were six factors that negatively impacted both HIBF and breastfeeding >6 months: Aboriginal/Torres Strait Islander ethnicity, previous birth caesarean section, previous preterm birth, smoking, obesity and a history of mental health illness (Table 3). Smoking conferred the greatest reduction in odds of HIBF (aOR 0.31; 95%CI 0.21–0.47) and being of Aboriginal/Torres Strait ethnicity the most reduced odds of breastfeeding >6 months when compared to White/European ethnicity (aOR 0.31; 95%CI 0.19–0.50) (Table 3).

Women who had a caesarean section birth had 22% reduced odds to breastfeed at high intensity (aOR 0.78; 95%CI 0.67–0.91; *p* < 0.01) and they were less likely to breastfeed >6 months (aOR 0.85; 95%CI 0.74–0.97, *p* = 0.02). A history of preterm birth was associated with 39% reduced odds of HIBF (aOR 0.61; 95%CI 0.48–0.77, *p* < 0.01) (Table 2) and reduced odds of breastfeeding >6 months (aOR 0.76; 95%CI 0.60–0.95, *p* = 0.02).

Compared to women with a healthy BMI (18.5–24.9 kg/m^2^), a high BMI ≥ 30 kg/m^2^ negatively impacted breastfeeding, with 38% reduced odds of HIBF (aOR 0.62; 95%CI 0.52–0.73 *p* < 0.01) and 31% reduced odds of breastfeeding >6 months (aOR 0.69; 95%CI 0.57–0.78, *p* < 0.01). The other previous pregnancy characteristic to negatively impact breastfeeding was a history of mental illness, with lower odds of both HIBF (aOR 0.69; 95%CI 0.58–0.83, *p* < 0.01) and breastfeeding >6 months (aOR 0.78; 95%CI 0.65–0.91, *p* < 0.01).

South Asian ethnicity was the only factor positively associated with both improved HIBF and breastfeeding >6 months. Compared to White/European ethnicity, women who identified as South Asian had 65% increased odds of HIBF (aOR 1.65; 95%CI 1.36–2.00, *p* < 0.01) and greater odds of breastfeeding >6 months (aOR 1.81; 95%CI 1.52–2.15, *p* < 0.01).

### 3.4. Factors Only Associated with Breastfeeding >6 Months

Three characteristics were negatively associated with duration of breastfeeding but not intensity; age <25 years compared to the 25–34 age grouping (0.74 aOR; 95%CI 0.58–0.94, *p* = 0.01), Middle Eastern ethnicity compared to White/European ethnic groupings (aOR 0.79; 95%CI 0.66–0.94, *p* = 0.01) and a BMI in an overweight range compared to a healthy BMI (aOR 0.86; 95%CI 0.75–0.99, *p* = 0.04). Other positively associated factors only identified for breastfeeding duration >6 months but not HIBF were for participants over 35 years age (1.25 95%CI 1.10–1.42, *p* < 0.01) or >40 years (1.52 95%CI 1.20–1.94, *p* < 0.01) compared to 25–34-year-old age groupings. Women who identified as South-East Asian ethnicity were also more likely to breastfeed >6 months (aOR 1.27; 95%CI 1.04–1.54, *p* = 0.02) compared to White/European participants. Compared to Australian-born status, migrant status had no association with HIBF; however, migrant status was positively associated with breastfeeding >6 months. Compared to Australian-born women, new migrants who had lived in Australia <5 years had 60% increased odds of breastfeeding >6 months (aOR 1.60; 95%CI 1.32–1.94, *p* < 0.01). All other associations are detailed in Table 3.

## 4. Discussion

This is the first study we are aware of that has found that optimal breastfeeding patterns can have direct metabolic benefits in a subsequent pregnancy. Potentially, this may reduce both maternal and infant morbidity. In a large, ethnically diverse population, our study has found that women who breastfed at a high intensity (exclusive or mostly breastfeeding) had improved odds of a normal fasting glucose in their subsequent pregnancy. We identified maternal characteristics that differed with length and intensity of breastfeeding, providing a more comprehensive understanding of women’s postpartum breastfeeding behaviour. Targeting factors to improve breastfeeding intensity may be one key to improved cardiometabolic health in subsequent pregnancies. Specific groups in this study who were vulnerable to suboptimal breastfeeding intensity and duration include women with a history of preterm birth, women with a high BMI, those who had a history of a mental health illness, women who identify as Aboriginal/Torres Strait Islander and smokers.

Women who do not meet the criteria for a diagnosis of GDM may still have some degree of dysglycaemia or gestational glucose intolerance (GGI) that places them at greater risk of type 2 diabetes [26]. Berekowsky et al. and others have found that an abnormal fasting OGTT was the value most correlated with a future risk of type 2 diabetes compared to OGTT results at one and two hours [27,28]. Other research has also found an abnormal fasting OGTT result alone predicts increased risk of a large for gestational age baby and other adverse pregnancy outcomes [29,30]. Supporting HIBF for all women may therefore reduce their risk for both adverse perinatal outcomes in a subsequent pregnancy and improve maternal long-term cardiometabolic risk.

Postpartum HIBF has been described as potentially an essential part of an endocrine reset process after a pregnancy-induced insulin resistant state [31]. Other research has found that compared to mixed or mostly formula infant feeding, it is HIBF that confers the most benefit for improved postpartum insulin resistance and lipid profiles and may underpin the long-term improved risk for metabolic disorders associated with breastfeeding [32]. The global burden of cardiometabolic disease requires a united and multi-faceted approach to complex drivers. This research identifies women with known risk factors for cardiometabolic disease, such as obesity, smoking and preterm birth, as also vulnerable to suboptimal breastfeeding intensity and duration and subsequent higher odds of failing their fasting pregnancy OGTT. The cumulative impact of existing cardiometabolic risk and poor breastfeeding needs addressing as one of the many drivers for global cardiometabolic disease burden [1,2,3]. Our study provides further evidence of the importance that early exclusive or mostly breastfeeding may be vital for long-term cardiometabolic health and crucial for an endocrine ‘reset’ postpartum. Routinely collected breastfeeding data inclusive of intensity information via tools such as the Sydney BLISS check will assist in uncovering drivers for optimal health outcomes for women.

Breastfeeding support programs are effective to improve breastfeeding and generally acceptable to women [33]. However, minimal research has integrated lifestyle programs with breastfeeding support interventions aimed at reducing type 2 diabetes risk [34]. A recent Cochrane Review found that at least 4–8 postpartum lactation support contacts were required to improve breastfeeding rates. Targeted support such as this is particularly important for populations found in this research [33]. If the WHO Sustainable Development Goal to reduce global NCD is to be met, all health promotion avenues should be pursued. Supporting breastfeeding must be included. Understanding the metabolic impact during pregnancy of previous breastfeeding duration and exclusivity, as well as identifying groups at risk for suboptimal breastfeeding, will assist in designing targeted interventions to improve lifetime cardiometabolic risk.

A limitation of this study is that there was no available information on some factors that may influence both breastfeeding and dysglycaemia. These factors include lack of available information in routine data on education level, exercise, diet, interpregnancy weight gain, time period between pregnancies or important baseline metabolic risk factors including lipid levels. The only available weight was for the current pregnancy. Potentially, there are factors such as exercise that participants who breastfeed for a greater duration are less likely to engage in.

Another limitation was the missing BLISS and OGTT values in the routine data. The COVID-19 pandemic may have influenced women attending to routine OGTT investigations. Local research found that some women who were pregnant experienced fear of public places due to the COVID-19 pandemic, and this, as well as lockdowns, may have contributed to reduced attendance for pregnancy OGTTs [35]. However, noncompliance with testing has been documented in various populations to be from 10–50% of women; therefore our missing OGTT data is consistent with other population-level data [36]. The missing BLISS should not be biased by patient selection, but was impacted by slow implementation due to staff shortages and lack of time to complete the new breastfeeding triage tool. Women who had chosen to formula feed in their previous pregnancy and did not breastfeed at all were often not given a BLISS score, and therefore our results do not fully capture the lowest intensity of no breastfeeding.

A strength of this cohort was the diversity of ethnicity, and therefore the results are potentially more applicable to other culturally mixed populations. The use of routine clinical data of both duration and intensity of breastfeeding for the entire health district was also a strength resulting in a large sample size. The Sydney BLISS check is applied to a non-selected clinical care population that is reflective and generalizable, as the participants were not a motivated breastfeeding research cohort.

The high-intensity BLISS check scoring is affirmed as a valid cut-off score via the association with greater duration of breastfeeding [37]. In this study, when compared to the LIBF group, we found HIBF in the first three months postpartum was associated with a 9-month greater median breastfeeding duration. Further highlighting that the fourth trimester postpartum period is crucial to provide breastfeeding support to optimise greater duration of breastfeeding and associated benefits to women and babies.

Another strength of the Sydney BLISS is the validation of the HAPO cut-off value of 5.1 mmol/L for fasting glucose in our ethnically diverse population. When our results were disaggregated by low and high intensity and glucose levels along a continuum, HIBF participants clearly diverged from the LIBF group at the IADPSG fasting normal cut-off point [38]. Higher IADPSG cut-off values are associated with increased obstetric morbidity, HIBF therefore may potentially be association with lower obstetric morbidity that will need further investigation.

In Australia, nearly 40% of women who do not breastfeed state ‘unsuccessful’ previous experience as the reason [39]. Successful implementation of the antenatal Sydney BLISS check in the study health district assists in addressing this issue for multiparous women, as they are given the opportunity for antenatal referral to a new telehealth lactation consultant clinic. Promoting and protecting breastfeeding for short- and long-term metabolic health, as well as infant health, requires population-level data to underpin appropriate support that the Sydney BLISS check can provide.

## 5. Conclusions

The objectives of this study were to understand, in an unselected culturally diverse population, if breastfeeding duration and intensity was associated with improved OGTT results in a subsequent pregnancy and identify specific groups of women who may be susceptible to suboptimal breastfeeding. This study provides information not only on the important gains of optimal breastfeeding for next pregnancy glycaemic control, but also identifies vulnerable breastfeeding populations to drive targeted intervention programs. During the pandemic era with constraints on health budgets and staffing, it has been difficult to provide evidence-based lactation support. Breastfeeding duration is vital for infant and maternal health, but this study has found the importance of mostly or exclusively breastfeeding as a public health issue to reduce GDM risk in a subsequent pregnancy and potentially improve long-term health.

## Figures and Tables

**Table 1:** Cohort characteristics by exposure of breastfeeding duration and intensity (*N* = 5374).

Maternal Characteristics	Duration of Breastfeeding Median Months (IQR)	*p* Value	High-Intensity Breastfeeding (*n* = 4074, 75.8%)	Low-Intensity Breastfeeding (*n* = 1300, 24.2%)	*p* Value
Maternal age		<0.001			<0.001
<25	6 (2–13)		69.46% (232)	30.54% (102)	
25–34	8 (4–15)		74.30% (1772)	25.70% (613)	
35–39	11 (6–18)		77.96% (1765)	22.04% (499)	
>39	12 (6–18)		78.01% (305)	21.99% (86)	
Ethnicity		<0.001			<0.001
South Asian	12 (6–18)		81.73% (1302)	18.27% (291)	
Southeast Asian	10 (5–14)		74.35% (661)	25.65% (228)	
White/European	9 (4–14)		73.09% (690)	26.91% (254)	
Middle Eastern	7 (3–14)		71.73% (789)	28.27% (311)	
Aboriginal and Torres Strait Islander	4 (2–9)		58.14% (50)	41.86% (36)	
Other	8 (4–14)		76.38% (582)	23.62% (180)	
Migrant status		<0.001			<0.001
<5 years	12 (6–18)		78.68% (1011)	21.32% (274)	
5–10 years	12 (6–18)		78.57% (759)	21.43% (207)	
>10 years	9 (4–15)		75.64% (798)	24.36% (267)	
Australian-born	7 (3–14)		71.31% (1213)	28.59% (488)	
Socioeconomic status		<0.001			<0.001
Least advantaged Q1	8 (3–15)		71.28% (1184)	28.72% (477)	
Q2	9 (4–14)		75.83% (549)	24.17% (175)	
Q3	11 (6–18)		77.56% (923)	22.44% (267)	
Q4	10 (5–17)		80.09% (531)	19.91% (132)	
Most advantaged Q5	11 (5–16)		78.06% (715)	21.94% (201)	
Parity		0.006			0.024
1	10 (4–17)		74.63% (2544)	25.37% (865)	
2–3	8 (4–15)		78.10% (1323)	21.90% (371)	
≥4	8 (4–18)		76.38% (207)	23.62% (64)	
Current pregnancy					
High risk of GDM *	9 (4–16)	0.859	74.61% (1740)	25.39% (592)	0.073
Lower risk of GDM	10 (4–16)		76.73% (2334)	23.27% (708)	
Smoking	4 (2–12)	<0.001	47.06% (48)	52.94% (54)	<0.001
No smoking	10 (5–17)		76.80% (2655)	23.20% (802)	
Comorbidities					
Polycystic ovary syndrome	8 (4–16)	0.23	73.11% (223)	26.89% (82)	0.26
No polycystic ovary syndrome	10 (4–16)		75.97% (3850)	24.03% (1218)	
History of mental health issue	7 (3–14)	<0.001	68.39% (541)	31.61% (250)	<0.001
No history of mental health issue	10 (4–18)		77.08% (3532)	22.92% (1050)	
History of hypertension	8 (4–14)	0.071	71.53% (206)	28.47% (82)	0.081
No history of hypertension	10 (4–16)		76.05% (3867)	23.95% (1218)	
BMI kg/m^2^		<0.001			<0.001
<18.5	8 (4–14)		82.94% (141)	17.06% (29)	
18.5–24.9	11 (5–17)		77.77% (1907)	22.23% (545)	
25.0–29.9	9 (4–17)		77.04% (1258)	22.96% (375)	
≥30.0	7 (3–15)		68.63% (768)	31.37% (351)	
Previous pregnancy complications					
History of GDM	9 (4–16)	0.635	74.96% (518)	25.04% (173)	0.578
No history of GDM	9 (4–16)		75.93% (3556)	24.07% (1127)	
Previous birth: caesarean section	9 (4–18)	0.851	72.94% (949)	27.06% (352)	0.006
Previous birth: vaginal	9 (4–16)		76.72% (3125)	23.28% (948)	
Previous birth: preterm	7 (4–14)	0.003	67.89% (258)	32.11% (122)	<0.001
Previous birth term	10 (4–17)		76.41% (3816)	23.59% (1178)	

GDM, gestational diabetes mellitus. * High-risk GDM criteria: previous GDM, age > 40, family history of diabetes, BMI > 35, previous large for gestation baby, polycystic ovary disease, corticosteroid medication use.

**Table 2:** Association between breastfeeding (BF) in a previous pregnancy and odds ratio (OR) of a gestational diabetes (GDM) diagnosis and/or of an abnormal elevated oral glucose tolerance test (OGTT) blood glucose result in current pregnancy. Total cohort *n* = 5374. High-intensity breastfeeding *n* = 4074.

**(a). High-Intensity Breastfeeding (HIBF)–Mostly or Exclusively Breastfeeding.**							
**Outcome**	**Cohort**	**HIBF** **% (*n*)**	**LIBF** **% (*n*)**	**Unadjusted** **OR (95%CI)**	***p***	**Adjusted** **OR (95%CI)**	***p***
Elevated Fasting OGTT	Total Cohort	5.99% (243)	8.02% (104)	0.53 (0.38, 0.73)	<0.01	0.53 (0.38, 0.75)	<0.01
	High Risk	8.93% (154)	12.24% (72)	0.56 (0.38, 0.83)	0.01	0.55 (0.36, 0.83)	0.01
	Low Risk	3.81% (89)	4.52% (32)	0.47 (0.27, 0.83)	0.01	0.48 (0.27, 0.86)	0.01
Elevated 1 h OGTT	Total Cohort	10.72% (376)	8.83% (100)	1.14 (0.85, 1.52)	0.37	1.20 (0.89, 1.63)	0.23
	High Risk	16.43% (236)	14.57% (73)	1.04 (0.73, 1.47)	0.84	1.05 (0.72, 1.52)	0.81
	Low Risk	6.75% (140)	4.28% (27)	1.52 (0.90, 2.59)	0.12	1.58 (0.92, 2.71)	0.09
Elevated 2 h OGTT	Total Cohort	10.38% (420)	10.78% (139)	0.96 (0.73, 1.26)	0.77	1.01 (0.76, 1.33)	0.97
	High Risk	16.07% (276)	15.92% (93)	0.91 (0.66, 1.26)	0.57	0.92 (0.65, 1.29)	0.62
	Low Risk	6.18% (144)	6.52% (46)	1.18 (0.73, 1.93)	0.50	1.23 (0.75, 2.01)	0.42
Diagnosed GDM	Total Cohort	18.09% (737)	18.77% (244)	0.87 (0.73, 1.03)	0.11	0.91 (0.75, 1.10)	0.34
	High Risk	26.72% (465)	27.87% (165)	0.86 (0.68, 1.07)	0.18	0.84 (0.65, 1.08)	0.18
	Low Risk	11.65% (272)	11.16% (79)	0.95 (0.71, 1.27)	0.75	1.02 (0.75, 1.37)	0.92
**(b). Breastfeeding duration as a continuous variable and elevated glucose.**							
**Outcome**	**Cohort**	**Normal** **Glucose** **BF Months (IQR)**	**Elevated** **Glucose** **BF months (IQR)**	**Unadjusted** **OR (95%CI)**	***p***	**Adjusted** **OR (95%CI)**	***p***
Fasting OGTT	Total Cohort	9 (4–16)	11 (4–18)	1.03 (1.01, 1.04)	<0.01	1.02 (1.00, 1.04)	0.04
	High Risk	9 (4–16)	9 (4–18)	1.02 (1.00, 1.04)	0.02	1.02 (0.99, 1.04)	0.15
	Low Risk	9 (4–16)	12 (6–19)	1.04 (1.01, 1.07)	0.01	1.03 (0.99, 1.06)	0.06
1 h OGTT	Total Cohort	9 (4–16)	12 (6–18)	1.02 (1.00, 1.03)	0.01	1.01 (0.99, 1.02)	0.34
	High Risk	9 (4–15)	11.5 (6–18)	1.02 (1.00, 1.04)	0.02	1.05 (0.72, 1.52)	0.81
	Low Risk	9 (4–16)	12 (6–18)	1.01 (0.99, 1.04)	0.23	1.00 (0.98, 1.03)	0.84
2 h OGTT	Total Cohort	9 (4–16)	11 (5–18)	1.01 (1.00, 1.02)	0.12	1.00 (0.99, 1.02)	0.86
	High Risk	9 (4–15)	11 (5–18)	1.02 (1.00, 1.04)	0.01	1.01 (0.99, 1.03)	0.62
	Low Risk	9 (4–16)	11 (5–18)	0.99 (0.97, 1.02)	0.50	0.98 (0.96, 1.01)	0.14
GDM	Total Cohort	9 (4–16)	11 (5–18)	1.02 (1.01, 1.02)	<0.01	1.01 (0.99, 1.02)	0.06
	High Risk	9 (4–16)	11 (5–18)	1.02 (1.00, 1.03)	0.01	1.01 (0.99, 1.02)	0.15
	Low Risk	9 (4–16)	12 (5–18)	1.02 (1.00, 1.03)	0.03	1.01 (0.99, 1.02)	0.21

High risk = high risk for GDM; previous GDM, maternal age ≥ 40 years, family history diabetes, BMI >35 kg/m^2^, previous baby with birth weight >4500 g or >90th centile, polycystic ovarian syndrome and current use of corticosteroids or antipsychotics. Adjusted covariables: maternal age, ethnicity, migrancy, socioeconomic status, BMI, parity, history of mental illness, history of hypertension, history of GDM and smoking during pregnancy. GDM, gestational diabetes diagnosis AIDPSG criteria, one or more values ≥ thresholds of fasting plasma glucose of 5.1 mmol/L and/or a 2 h plasma glucose level of 8.5 mmol/L following a 75 g OGTT. HIBF, high-intensity breastfeeding; LIBF, lower intensity breastfeeding.

**Table 3:** Factors Associated with High-intensity Breastfeeding and Breastfeeding Duration >6 Months by Maternal Characteristics. Total cohort *N* = 5374. High-intensity breastfeeding *n* = 4074. Breastfeeding >6 months *n* = 3222.

Characteristic	High-Intensity Breastfeeding				Breastfeeding Duration > 6 Months			
	Unadjusted OR (95%CI)	*p*	aOR (95%CI)	*p*	Unadjusted OR (95%CI)	*p*	aOR (95%CI)	*p*
Maternal age								
<25	0.79 (0.61, 1.01)	ns	0.95 (0.73, 1.23)	ns	0.62 (0.49, 0.78)	<0.01	0.74 (0.58, 0.94)	0.01
25–34	Ref	-	-	-	Ref	-	-	-
35–39	1.22 (1.07, 1.40)	0.01	1.10 (0.96, 1.27)	ns	1.40 (1.24, 1.58)	<0.01	1.25 (1.10, 1.42)	<0.01
40+	1.23 (0.95, 1.59)	ns	1.17 (0.89, 1.53)	ns	1.53 (1.22, 1.93)	<0.01	1.52 (1.20, 1.94)	<0.01
Ethnicity								
South Asian	1.65 (1.36, 2.00)	<0.01	1.65 (1.36, 2.00)	<0.01	1.81 (1.52, 2.15)	<0.01	1.81 (1.52, 2.15)	<0.01
South-East Asian	1.07 (0.87, 1.31)	ns	1.07 (0.87, 1.31)	ns	1.27 (1.04, 1.54)	0.02	1.27 (1.04, 1.54)	0.02
White/European	Ref	-	-	-	Ref	-	-	-
Middle Eastern	0.93 (0.77, 1.13)	ns	0.93 (0.77, 1.13)	ns	0.79 (0.66, 0.94)	0.01	0.79 (0.66, 0.94)	0.01
Aboriginal/Torres Strait Islander	0.51 (0.33, 0.80)	0.01	0.51 (0.33, 0.80)	0.01	0.31 (0.19, 0.50)	<0.01	0.31 (0.19, 0.50)	<0.01
Other	1.19 (0.95, 1.48)	ns	1.19 (0.95, 1.48)	ns	0.97 (0.79, 1.18)	ns	0.97 (0.79, 1.18)	ns
Migrant status								
<5 years	1.48 (1.25, 1.76)	<0.01	1.20 (0.97, 1.48)	ns	1.94 (1.66, 2.26)	<0.01	1.60 (1.32, 1.94)	<0.01
5–10 years	1.48 (1.22, 1.78)	<0.01	1.18 (0.94, 1.47)	ns	1.95 (1.65, 2.31)	<0.01	1.56 (1.27, 1.91)	<0.01
>10 years	1.25 (1.05, 1.49)	0.01	1.10 (0.90, 1.35)	ns	1.51 (1.29, 1.77)	<0.01	1.32 (1.09, 1.59)	0.01
Australian-born	Ref	-	-	-	Ref	-	-	-
Previous complications								
History of GDM	0.95 (0.79, 1.14)	ns	0.92 (0.76, 1.11)	ns	1.05 (0.88, 1.24)	ns	0.97 (0.82, 1.15)	ns
History of hypertension	0.79 (0.61, 1.03)	ns	0.88 (0.68, 1.15)	ns	0.90 (0.70, 1.14)	ns	1.03 (0.80, 1.33)	ns
Previous birth caesarean section	0.82 (0.71, 0.94)	0.01	0.78 (0.67, 0.91)	<0.01	0.93 (0.82, 1.06)	ns	0.85 (0.74, 0.97)	0.02
Previous birth preterm	0.65 (0.52, 0.82)	<0.01	0.61 (0.48, 0.77)	<0.01	0.73 (0.59, 0.91)	0.01	0.76 (0.60, 0.95)	0.02
Current pregnancy								
Smoking	0.27 (0.18, 0.40)	<0.01	0.31 (0.21, 0.47)	<0.01	0.33 (0.22, 0.49)	<0.01	0.44 (0.29, 0.67)	<0.01
BMI kg/m^2^								
<18.5	1.39 (0.92, 2.10)	ns	1.53 (1.01, 2.32)	ns	0.87 (0.63, 1.21)	ns	0.96 (0.69, 1.33)	ns
18.5–24.9	Ref	-	-	-	Ref	-	-	-
25.0–29.9	0.96 (0.83, 1.11)	ns	0.89 (0.77, 1.04)	ns	0.87 (0.76, 0.99)	0.04	0.86 (0.75, 0.99)	0.04
≥30.0	0.63 (0.53, 0.73)	<0.01	0.62 (0.52, 0.73)	<0.01	0.59 (0.51, 0.68)	<0.01	0.69 (0.57, 0.78)	<0.01
History of mental health issue	0.64 (0.55, 0.76)	<0.01	0.69 (0.58, 0.83)	<0.01	0.67 (0.57, 0.78)	<0.01	0.78 (0.65, 0.91)	<0.01

GDM = gestational diabetes. ns = not statistically significant. Ref = reference. Multivariate model may adjusted for: age, ethnicity, time in Australia, SES, BMI, parity, history of mental illness, history of hypertension, history of GDM, history of caesarean section, history of preterm birth, unplanned pregnancy, smoking.

## Data Availability

The dataset generated during the current study are not publicly available due to institutional restrictions on patient data but are available from the corresponding author upon reasonable request and institutional approval.

## Supplementary Materials

The following supporting information can be downloaded at: https://www.mdpi.com/article/10.3390/nu16010028/s1, File S1. Sydney BLISS check uptake by site; File S2. The Sydney BLISS check; File S3. Statistical notes confounding factors; File S4. Statistical notes missingness; File S5. Correlation breastfeeding length and intensity; File S6. Breastfeeding intensity and OGTT. References [25,40,41] are cited in supplementary file.
