# Supplementary material for: Previous High-Intensity Breastfeeding Lowers the Risk of an Abnormal Fasting Glucose in a Subsequent Pregnancy Oral Glucose Tolerance Test
Source: Nutrients. 2023 Dec 21;16(1):28. doi: 10.3390/nu16010028 (PMC10780944; doi:10.3390/nu16010028)
Supplement: Supplementary file 1 [file nutrients-16-00028-s001.zip › nutrients-2766401-supplementary.pdf]

Supplementary Materials

Previous high intensity breastfeeding lowers the risk of an abnormal fasting glucose in a subsequent pregnancy oral glucose tolerance test

Melov SJ, Elhindi J, White L, McNab J, Lee V, Donnolley K, Alahakoon TI, Padmanaban S, Cheung NW, Pasupathy D

File S1: Supplementary Figure S1. (Page 2)
Uptake of Breastfeeding Length Intensity Scoring System (BLISS) by site over time.

File S2: Breastfeeding Length Intensity Scoring System (Sydney BLISS check) (Page 3)

File S3: Statistical notes: Possible confounding factors that each exposure was adjusted for. (Page 4)

File S4: Statistical notes: More detailed explanation of the mechanisms behind missingness assumptions. (Page 5)

File S5: Supplementary Figure S2. (Page 6)
Descriptive statistics of the correlation between breastfeeding intensity and breastfeeding length.

File S6: Supplementary Figure S3. (Page 7)
Previous pregnancy breastfeeding and subsequent pregnancy fasting OGTT plasma glucose

File S1

Supplementary Figure S1: Implementation of breastfeeding length intensity score system (Sydney BLISS check) by Western Sydney Local Health District site over time.

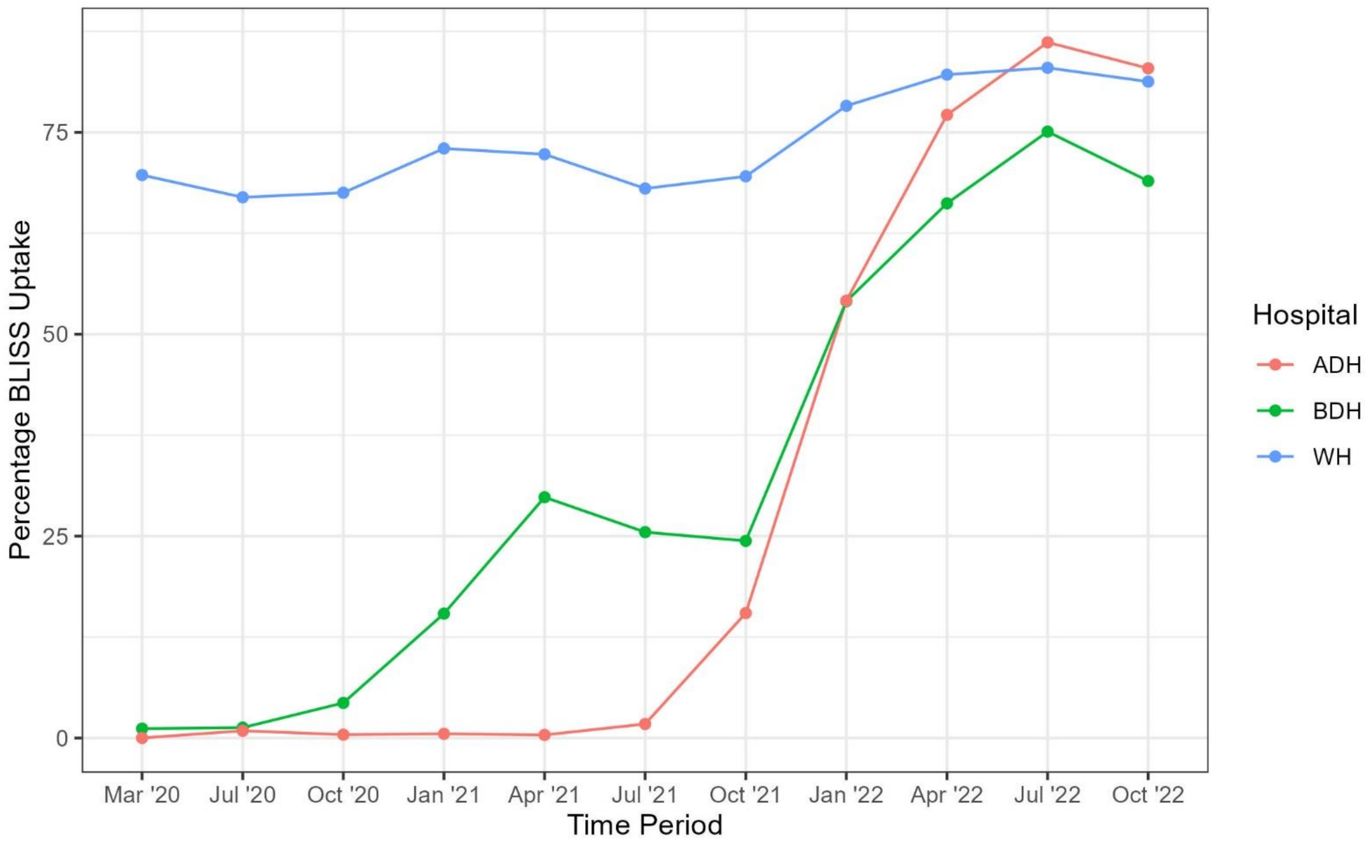

File S2: Breastfeeding Length Intensity Scoring System (Sydney BLISS check)

The Sydney BLISS check: Breastfeeding Length Intensity Scoring System

How many babies have you breastfed? (*circle*) 0 1 2 3 4 5 6 other____

Please add together all the months you breastfed each of your children.
For example if you have had two children and breastfed the first baby for 4 months and the second for 6 months this equals 10 months total lifetime breastfeeding.
Total months you have actually been breastfeeding all your children? ____Months

How old was your last baby when you stopped breastfeeding? Months____ Days___

1. During your last baby's **first week of life** how did you feed?

a. Breastfeeding only □ 6 c. Formula feeding only □ 0

b. Breastfeeding and formula □ 1

If you ticked **b.** **did you give:** **Only** 1-4 formula feeds in **first week** □ 4
Most **days** 1-3 formula feeds □ 2
Most **days** 4 or more formula feeds □ 1 __ / 6

2. During the **first month of life** how did you feed your last baby? (*after first week*)

a. Breastfeeding only □ 7 c. Formula feeding only □ 0
b. Breastfeeding and formula □ 1
If you ticked **b.** **did you give:** **Only** 1-4 formula feeds **in first month** □ 6
Most **weeks** only 1-4 formula □ 6
Most **days** 1-3 formula feeds □ 2
Most **days** 4 or more formula feeds □ 0 __ / 7

3. **After the first month** how did you feed your last baby?

a. Breastfeeding only □ 6 c. Formula feeding only □ 0
b. Breastfeeding and formula □ 1
If you ticked **b.** **did you give:** Most **weeks only 1-4** □ 5
Most **days** 1-3 formula feeds □ 2
Most **days** 4 or more formula feeds □ 0 __ / 6

4. **At three months** of age how did you feed your last baby?

a. Breastfeeding only □ 6 c. Formula feeding only □ 0
b. Breastfeeding and formula □ 1
If you ticked **b.** **did you give:** **Most weeks only 1-4 formula** □ 5
Most **days** 1-3 formula feeds □ 2
Most **days** 4 or more formula feeds □ 0 __ / 6

Are you currently breastfeeding? (circle) No Yes

Why did you stop breastfeeding your last baby? (*can tick more than one reason*)

1. Attachment or Feeding Problems □
2. Baby's Age □
3. Baby Lost Interest □
4. Baby unsettled □
5. Problem's with Baby's Weight gain □
6. Problem's with Expressing □
8. Low Supply of breast milk □
9. Mastitis □
10. Taking medication couldn't breastfeed □
11. Pregnant or planning pregnancy
12. Returning to work/study □
13. Other Reason__________________
____

BLISS Check version 1 7.2.2020

Developed by Sarah J Melov WSLHD ©

File S3: Possible confounding factors that each exposure was adjusted for.

They were, respectively, adjusted for:

- Maternal age: ethnicity, migrancy, SES, BMI, parity, history of hypertension, history of GDM, history of preterm birth, and smoking.
- Ethnicity: none.
- Migrancy: BMI, age, ethnicity, SES.
- History of GDM: age, ethnicity, BMI, parity.
- History of hypertension: age, ethnicity, BMI, history of GDM, smoking.
- Previous Birth CS: age, ethnicity, SES, BMI, parity, history of mental illness, history of hypertension, history of GDM, smoking, migrancy.
- Previous Birth PTB: age, ethnicity, SES, BMI, parity, history of mental illness, history of hypertension, history of GDM, smoking, migrancy.
- Smoking: age, ethnicity, SES, parity, history of mental illness.
- BMI: age, ethnicity, migrancy, parity, history of mental illness.
- History of mental illness: age, ethnicity, SES.

Statistic reference:
Daniel Westreich, Sander Greenland, The Table 2 Fallacy: Presenting and Interpreting Confounder and Modifier Coefficients, American Journal of Epidemiology, Volume 177, Issue 4, 15 February 2013, Pages 292–298, https://doi.org/10.1093/aje/kws412.

Bandoli G, Palmsten K, Chambers CD, Jelliffe-Pawlowski LL, Baer RJ, Thompson CA. Revisiting the Table 2 fallacy: A motivating example examining preeclampsia and preterm birth. Paediatr Perinat Epidemiol. 2018 Jul;32(4):390-397. doi: 10.1111/ppe.12474. Epub 2018 May 21. PMID: 29782045; PMCID: PMC6103824.

File S4: More detailed explanation of the mechanisms behind missingness assumptions.

The primary independent variable (breastfeeding intensity) and dependent variable (glucose tolerance) had non-negligible levels of missingness. Neither had substantial levels of missingness. We believe that two mechanisms plausibly explain the missingness of the breastfeeding intensity variable:

1. Women that did not breastfeed in their previous pregnancy were not given a breastfeeding intensity score.

2. The breastfeeding intensity score was a non-essential booking item. On busy or understaffed days, it may not have been collected, particularly during pandemic-related changing procedures for midwives at booking.

The first mechanism is of greater concern and has been addressed as a limitation of the implementation procedure to date. Importantly, the mechanism (no breastfeeding) is related to the missing variable (breastfeeding intensity), so the missing not at random (MNAR) assumption is plausible. When the MNAR assumption is plausible, imputation methods are not appropriate, as the missingness cannot be modelled explicitly. [Ref 1]

We believe that two mechanisms plausibly explain the missingness of the glucose tolerance variable:

1. The glucose tolerance values are a non-essential booking item. In many instances, the attending clinician preferred to record "GDM" or "No GDM" instead of the precise values.

2. Some women were not administered a glucose tolerance test, such as those that could not tolerate the glucose drink or those that booked later in pregnancy.

Whilst the first mechanism suggests that the missing completely at random (MCAR) assumption may be plausible, it is difficult to assess an MCAR hypothesis and most believe that in practice it is rarely plausible. [Ref 1]

The second mechanism is difficult to unpack, as the reasons women do not attend the recommended OGTT or are not able to tolerate the glucose drink are varied and unknown particularly during the pandemic, and therefore so is their relationship with the missing variable (glucose tolerance test result). As a consequence, some instances of the second mechanism may be MNAR whereas some may be missing at random (MAR). Each MAR instance would demand its own imputation model as the factors predictive of one reason for not tolerating the glucose drink may differ from another.

We did not implement a worst–best sensitivity analysis as we believe that the envelope of results it would articulate would be too wide to sensibly interpret. Such an analysis would involve assigning those with missing breastfeeding intensity the best/worst score, those with missing glucose tolerance values the best/worst result (two standard deviations above or below the mean, for example), and permutations thereof. However, that would produce four uniquely 'extreme' analyses that would be difficult to corroborate.

Reference 1: Jakobsen, J.C., Gluud, C., Wetterslev, J. et al. When and how should multiple imputation be used for handling missing data in randomised clinical trials – a practical guide with flowcharts. BMC Med Res Methodol 17, 162 (2017). https://doi.org/10.1186/s12874-017-0442-1.

File S5: Supplementary Figure S2.
Descriptive statistics of the correlation between breastfeeding intensity and breastfeeding length.

The raw breastfeeding intensity score is not normally distributed. Therefore, Spearman's rank correlation coefficient is used to estimate the correlation between breastfeeding length and intensity.

The estimated coefficient is 0.50 with a 95% confidence interval of 0.48 to 0.52 and a p-value < 0.01.

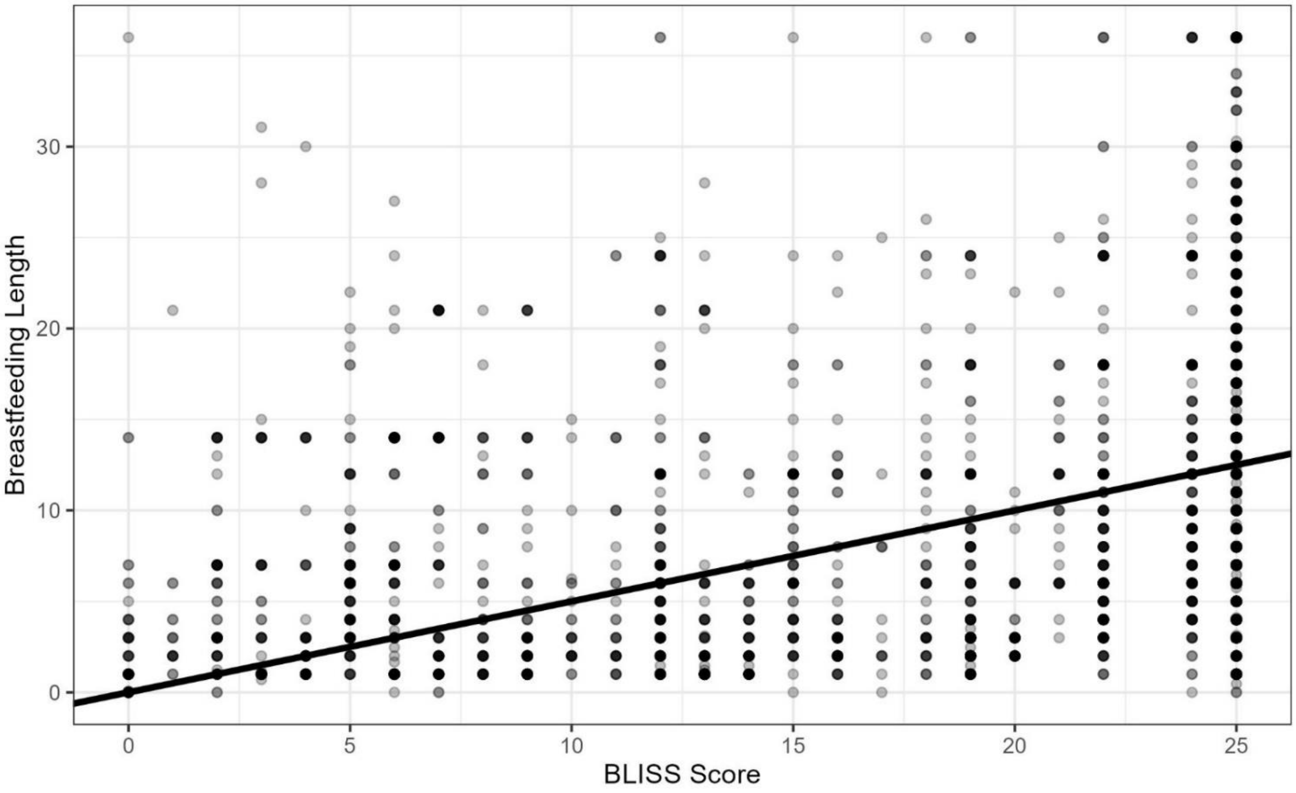

When we partition by high (HIBF) and lower intensity breastfeeding (LIBF), we observe:

- HIBF has median length of 12 months and IQR of 6 – 18 months.
- LIBF has median length of 3 months and IQR of 1 – 7 months.
- This difference is statistically significant (p < 0.01).

File S6. Supplementary Figure S3.
Previous pregnancy breastfeeding and subsequent pregnancy fasting OGTT plasma glucose

1. Lower intensity breastfeeding (BLISS 1-18) *n* = 1,300
2. High intensity breastfeeding (BLISS 19-25) *n* = 4,074

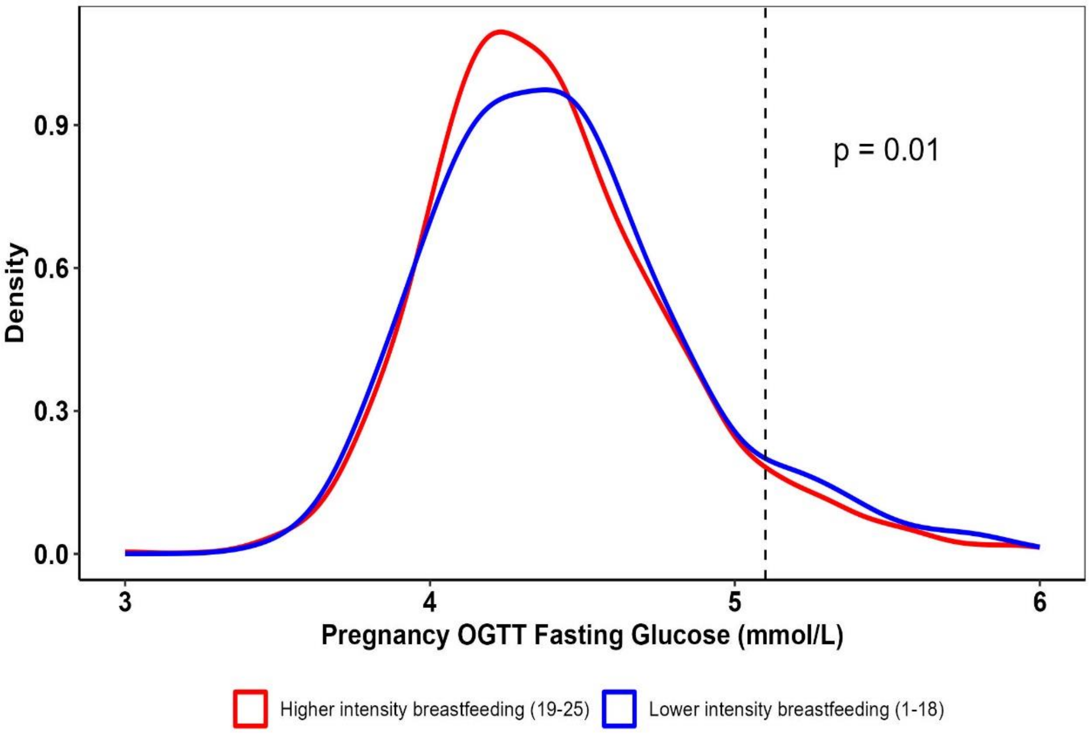

Kolmogorov–Smirnov test for the equality of two empirical distribution functions, with calculations based on a Monte Carlo simulation of 100,000 samples, returned a p-value of 0.01.
The observed separation of the two curves is statistically significant.
